# Hangeshashinto for prevention of oral mucositis in patients undergoing hematopoietic stem cell transplantation: a randomized phase II study

**DOI:** 10.1007/s00520-023-08175-7

**Published:** 2023-11-18

**Authors:** Masako Yoshimatsu, Yumiko Kawashita, Sakiko Soutome, Maho Murata, Yasushi Sawayama, Tadafumi Kurogi, Noriko Nakao, Yasushi Miyazaki, Masahiro Umeda, Takashi Ukai

**Affiliations:** 1 Oral Management Center, Nagasaki University Hospital, 1-7-1 Sakamoto, Nagasaki, 852-8501 Japan; 2 Department of Oral Health, Nagasaki University Graduate School of Biomedical Sciences, Nagasaki, Japan; 3 Department of Clinical Oral Oncology, Nagasaki University Graduate School of Biomedical Sciences, Nagasaki, Japan; 4 Department of Hematology, Japanese Red Cross Nagasaki Genbaku Hospital, Nagasaki, Japan; 5 Department of Hematology, Atomic Bomb Disease and Hibakusha Medicine Unit, Nagasaki University Graduate School of Biomedical Sciences, Nagasaki, Japan

**Keywords:** Hangeshashinto, Oral mucositis, Hematopoietic stem cell transplantation, Conditioning regimen

## Abstract

**Purpose:**

Oral mucositis (OM) is a side effect associated with cancer treatment. Hangeshashinto (HST), a Kampo medicine, was originally prescribed to treat diarrhea, gastritis, and stomatitis. Several reports have described the effects of HST for OM induced by chemotherapy in patients with gastric or colorectal cancer. In this study, the effects of HST for prevention of OM were investigated in patients undergoing hematopoietic stem cell transplantation (HSCT).

**Methods:**

Thirty patients scheduled to receive allogeneic grafts were enrolled from July 2020 to December 2021. They were randomly assigned to two groups and instructed to wash their mouth using HST dissolved in saline solution or using only saline solution three times a day. The observation period was from the initiation date of the conditioning regimen to the date of engraftment, and the end point was the incidence of OM.

**Results:**

Eighteen patients developed OM, the most severe of which was Grade (G)3. There was no significant difference in the incidence of OM between the HST group and the control group. However, a negative correlation tended to be observed between the duration using HST use and the duration of OM (G2–3: *P* = 0.027, G3: *P* = 0.047).

**Conclusions:**

The present study demonstrated that HST use did not clearly inhibit onset of OM but showed a tendency to inhibit OM exacerbation. However, further studies are necessary to fully understand the effects of HST on OM in patients undergoing HSCT.

**Trial registration:**

This study was registered in the Japan Registry of Clinical Trials on 7 May 2020 (jRCTs071200012).

## Introduction

Oral mucositis (OM) is a side effect associated with cancer treatment. The incidence of OM is 20% to 40% in patients undergoing conventional chemotherapy, 60% to 85% in those undergoing hematopoietic stem cell transplantation (HSCT), and almost 100% in those undergoing chemoradiation therapy for head and neck cancer [1–3]. OM can reduce patients’ quality of life because of pain-induced difficulty in eating, swallowing, talking, or brushing teeth. It sometimes causes discontinuation of cancer treatment. Patients with OM may also be susceptible to infection through the mucous membranes, and some outcomes are fatal [4–6].

Numerous reports focusing on prevention or treatment of OM secondary to cancer therapy have been published [7, 8]. Basic oral care by patients themselves or care providers is important for inhibition of OM. Educational interventions that help patients to understand the importance of oral care can also facilitate smooth completion of a series of treatments. According to the latest guidelines for management of OM by the Multinational Association of Supportive Care in Cancer and International Society of Oral Oncology [8], intraoral photobiomodulation is a rapidly growing field for patients who undergo HSCT. However, the guidelines also mention that photobiomodulation has carcinogenic effects; thus, the clinician must adequately inform patients before treatment. Oral cryotherapy for patients receiving high-dose melphalan as conditioning for autologous HSCT is beneficial for prevention of OM because of its vasoconstriction effect. In addition, the guidelines mention several antimicrobial, natural, and other agents. However, many patients with declining quality of life because of OM are still recognized in the clinical setting.

Chemotherapy-induced OM is associated with direct damage of mucosal tissues by cell death and the development of secondary oral infection due to bone marrow suppression [1, 9, 10]. The pathogenesis of OM is complicated and involves many inter-related factors. Reactive oxygen species are generated in the initiation phase of OM. Pro-inflammatory cytokines such as tumor necrosis factor-α, interleukin (IL)-6, IL-1β, and cyclooxygenase-2 are upregulated as OM progresses. Nuclear factor-κB, which upregulates these cytokines, also plays a key role in determining the severity of OM. In addition, decreased immune function and ulcerations associated with OM induce bacterial infections and cause intractable OM.

Hangeshashinto (HST), a Kampo medicine, was originally prescribed to treat diarrhea, gastritis, and stomatitis. HST contains seven extracted crude drugs (*Coptidis* Rhizoma, *Ginseng* Radix, *Glycyrrhizae* Radix, *Pinelliae* Tuber, *Scutellariae* Radix, *Zingiberis* Rhizoma Processum, and *Zizyphi* Fructus) and has multiple pharmacological effects [11, 12]. Although the effects of HST have been reported in patients who developed OM secondary to chemotherapy for gastric or colorectal cancer [13–15], its effects in HSCT patients receiving high-dose chemotherapy are unclear. This study was performed to evaluate the effects of HST for prevention OM in patients undergoing HSCT.

## Methods

### Patients and protocol design

This randomized phase II study was performed to investigate the effects of a mouthwash containing HST for prevention of OM in patients undergoing HSCT in Nagasaki University Hospital. The study protocol was approved by the Clinical Research Review Board of Nagasaki University on 28 April 2020 (#CRB7180001). Thirty patients scheduled to receive allogeneic grafts were enrolled from July 2020 to December 2021. After providing written informed consent, the patients underwent oral examinations and continuously received professional oral care interventions during data collection. They were randomly assigned to 2 groups of 15 patients each. Patients using HST were instructed to wash their mouth three times a day with 2.5 g HST (TJ-14; Tsumura, Tokyo, Japan) dissolved in 40 mL saline solution after brushing after meals. Patients assigned to the control group were instructed to wash their mouth using only saline solution. The duration of HST use was from initiation of the conditioning regimen of HSCT to the date of engraftment. Concomitant drugs were not restricted. If engraftment failure occurred more than 28 days after transplantation, data collection was finished.

### Data collection

The following patient characteristics were collected: age, sex, smoking history, drinking history, body mass index, performance status, diagnosis, conditioning regimen, donor, and duration from pretreatment to engraftment. The following oral findings and dental treatments prior to HSCT were also collected: number of remaining teeth, simplified debris index, state of alveolar bone loss, periodontal pocket depth, bleeding on probing, periapical periodontitis, high-grade caries, tooth extraction, and apicoectomy.

### Outcomes

The endpoint of this study was the incidence of OM. Oral assessment of each patient was performed by a single evaluator (M.Y.). The severity of OM was determined with reference to the National Cancer Institute Common Terminology Criteria for Adverse Events version 5.0 as follows: Grade (G)1, asymptomatic or mild symptoms, intervention not indicated; G2, moderate pain or ulcer that does not interfere with oral intake, modified diet indicated; G3, severe pain interfering with oral intake; G4, life-threatening consequences, urgent intervention indicated; and G5, death. Adverse events were recorded regardless of whether they had a causal link to the mouthwash used during the study period.

### Statistical analysis

Data are expressed as number of patients or median [25^th^–75^th^ percentile]. The cumulative incidence of OM was depicted using a Kaplan–Meier curve and analyzed with a log-rank test. The Mann–Whitney *U* test, Fisher’s exact test, the Kruskal–Wallis test, or Spearman’s rank correlation coefficient was used to evaluate the patient characteristics, and the occurrence and duration of OM. SPSS software version 27.0 (Japan IBM Co., Tokyo, Japan) was used for statistical analysis. All *P* values were based on a two-sided hypothesis, and *P* < 0.05 was considered statistically significant.

## Results

### Patient characteristics

The patients’ characteristics are summarized in Table 1. The median age was 56.0 years in the HST group and 58.0 years in the control group. Nineteen patients were male and 11 were female. The patients’ diagnoses were acute myeloid leukemia (*n* = 11), adult T-cell leukemia/lymphoma (*n* = 5), myelodysplastic syndromes (*n* = 4), acute lymphoid leukemia (*n* = 2), and other hematological diseases. The types of conditioning regimens were myeloablative conditioning (MAC) (*n* = 12) and reduced-intensity conditioning (RIC) (*n* = 18). Twenty-two patients received grafts from related donors, and eight received grafts from unrelated donors. The median duration from pretreatment to engraftment was 20.0 days in the HST group and 21.0 days in the control group. The median number of remaining teeth was 28.0 in the HST group and 26.0 in the control group, and 13 patients underwent tooth extraction or apicoectomy to prevent infection prior to the starting day of pretreatment. There was no significant difference in background factors between two groups.

**Table 1:** Patient characteristics

		HST	Control	*P* value
Age		56.0 [52.0–62.5]	58.0 [51.5–60.5]	0.902^§^
Sex	Male	10	9	0.705^†^
	Female	5	6	
Smoking	( −)	11	11	1.000^†^
	( +)	4	4	
Drinking	( −)	9	9	0.842^†^
	Socially	4	3	
	( +)	2	3	
Body mass index, kg/m^2^		22.2 [20.0–23.1]	19.9 [17.9–22.1]	0.217^§^
Performance status	0	5	2	0.107^†^
	1	8	13	
	2	2	0	
Diagnosis	AML	4	7	0.546^†^
	ATL	3	2	
	MDS	1	3	
	ALL	2	0	
	MDS + AML	1	0	
	MPAL	1	0	
	CMML	1	0	
	DLBCL	0	1	
	Others	2	2	
Conditioning regimen	MAC	6	6	1.000^†^
	RIC	9	9	
Donor	Related	13	9	0.215^†^
	Unrelated	2	6	
Duration from pretreatment to engraftment, days		20.0 [19.0–21.0]	21.0 [19.5–22.5]	0.285^§^
Number of remaining teeth		28.0 [24.5–28.0]	26.0 [22.0–27.0]	0.187^§^
Simplified debris index		0.33 [0.0–0.8]	0.33 [0.1–1.0]	0.539^§^
Alveolar bone loss ≥ 1/2		5	2	0.390^†^
Periodontal pocket depth ≥ 5 mm		3	4	1.000^†^
Bleeding on probing		10	9	0.705^†^
Periapical periodontitis ≥ 5 mm		3	5	0.682^†^
High-grade caries		2	4	0.651^†^
Tooth extraction / Apicoectomy		7	6	0.713^†^

Data are presented as number of patients or median [25–75% tale]

*HST* Hangeshashinto, *AML* acute myeloid leukemia, *ATL* adult T-cell leukemia/lymphoma, *MDS* myelodysplastic syndromes, *ALL* acute lymphoid leukemia, *MPAL* mixed phenotype acute leukemia, *CMML* chronic myelomonocytic leukemia, *DLBCL* diffuse large B-cell lymphoma, *MAC* myeloablative conditioning, *RIC* reduced-intensity conditioning

^§^Mann–Whitney *U* test, ^†^Fisher’s exact test

### Cumulative incidence of OM

**Fig. 1:**
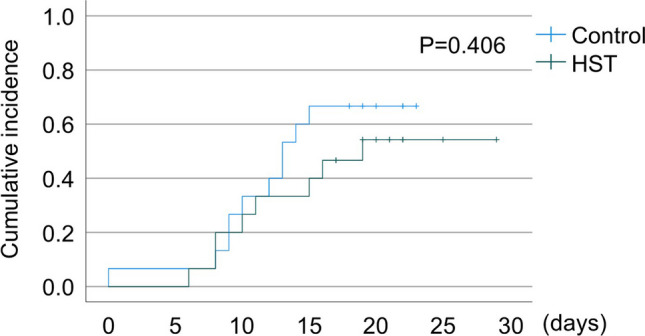
Cumulative incidence of oral mucositis (HST group vs. control group). HST, Hangeshashinto

Eight patients in the HST group and 10 patients in the control group developed OM. No patients developed G4 or G5 OM. Figure 1 shows the cumulative incidence of OM. There was no significant difference between the two groups (*P* = 0.406).

### Factors related to occurrence of OM

Table 2 shows the relationship of each factor with the occurrence of OM. The incidence of G1, G2, and G3 OM was significantly higher in patients who received the MAC regimen than in those who received the RIC regimen (G1 *P* < 0.001, G2 *P* = 0.001, G3 *P* = 0.002). There was no significant difference in the duration of HST use between patients with and without OM.

**Table 2:** Factors related to occurrence of oral mucositis

		G1		*P* value	G2		*P* value	G3		*P* value
		( −)	( +)		( −)	( +)		( −)	( +)	
Sex	Male	10	9	0.121^†^	12	7	1.000^†^	16	3	0.641^†^
	Female	2	9		7	4		8	3	
Smoking	( −)	9	13	1.000^†^	14	8	1.000^†^	17	5	1.000^†^
	( +)	3	5		5	3		7	1	
Drinking	( −)	8	10	0.605^†^	10	8	0.367^†^	13	5	0.297^†^
	Socially	3	4		6	1		7	0	
	( +)	1	4		3	2		4	1	
Performance status	0	3	4	0.933^†^	5	2	0.831^†^	6	1	0.527^†^
	1	8	13		13	8		17	4	
	2	1	1		1	1		1	1	
Conditioning regimen	MAC	0	12	< 0.001^†^	3	9	0.001^†^	6	6	0.002^†^
	RIC	12	6		16	2		18	0	
Donor	Related	10	12	0.419^†^	16	6	0.104^†^	19	3	0.300^†^
	Unrelated	2	6		3	5		5	3	
Alveolar bone loss ≥ 1/2	( −)	8	15	0.392^†^	13	10	0.215^†^	18	5	1.000^†^
	( +)	4	3		6	1		6	1	
Periodontal pocket depth ≥ 5 mm	( −)	9	14	1.000^†^	15	8	1.000^†^	19	4	0.603^†^
	( +)	3	4		4	3		5	2	
Bleeding on probing	( −)	3	8	0.442^†^	5	6	0.238^†^	8	3	0.641^†^
	( +)	9	10		14	5		16	3	
Periapical periodontitis ≥ 5 mm	( −)	9	13	1.000^†^	15	7	0.417^†^	18	4	0.645^†^
	( +)	3	5		4	4		6	2	
High-grade caries	( −)	9	15	0.660^†^	15	9	1.000^†^	20	4	0.571^†^
	( +)	3	3		4	2		4	2	
Tooth extraction / Apicoectomy	( −)	6	11	0.711^†^	10	7	0.708^†^	13	4	0.672^†^
	( +)	6	7		9	4		11	2	
Age		56 [49.5–64.0]	56.5 [52.0–61.3]	0.950^§^	57 [51.0–63.0]	56 [49.0–61.0]	0.832^§^	57 [51.5–62.5]	54 [48.3–61.3]	0.527^§^
Body mass index, kg/m^2^		21.0 [19.4–23.1]	20.5 [18.4–23.0]	0.819^§^	20.7 [19.2–23.1]	21.4 [18.2–22.9]	0.866^§^	20.8 [19.3–23.1]	20.7 [15.5–23.4]	0.631^§^
Duration from pretreatment to engraftment, days		20.5 [19.0–22.8]	20 [19.0–21.0]	0.723^§^	21 [19.0–22.0]	20 [19.0–24.0]	0.832^§^	20 [19.0–21.8]	20.5 [19.8–27.0]	0.402^§^
Number of remaining teeth		28 [23.3–29.5]	26.5 [21.0–27.3]	0.200^§^	27 [24.0–28.0]	26 [20.0–27.0]	0.123^§^	27 [21.5–28.0]	26.5 [22.0–27.3]	0.527^§^
Simplified debris index		0.50 [0.21–1.0]	0.17 [0.0–1.0]	0.267^§^	0.33 [0.0–1.0]	0.17 [0.0–1.0]	0.703^§^	0.33 [0.0–1.0]	0.59 [0.0–1.5]	0.667^§^
Duration of HST use, days		5.5 [0.0–19.5]	0 [0.0–18.0]	0.491^§^	4 [0.0–20.0]	0 [0.0–3.0]	0.094^§^	4 [0.0–19.5]	0 [0.0–0.75]	0.065^§^

Data are presented as number of patients or median [25–75% tale]

*G* grade, *MAC* myeloablative conditioning, *RIC* reduced-intensity conditioning, *HST* Hangeshashinto

^†^Fisher’s exact test, ^§^Mann–Whitney *U* test

### Relationship between each variable and duration of OM

**Fig. 2:**
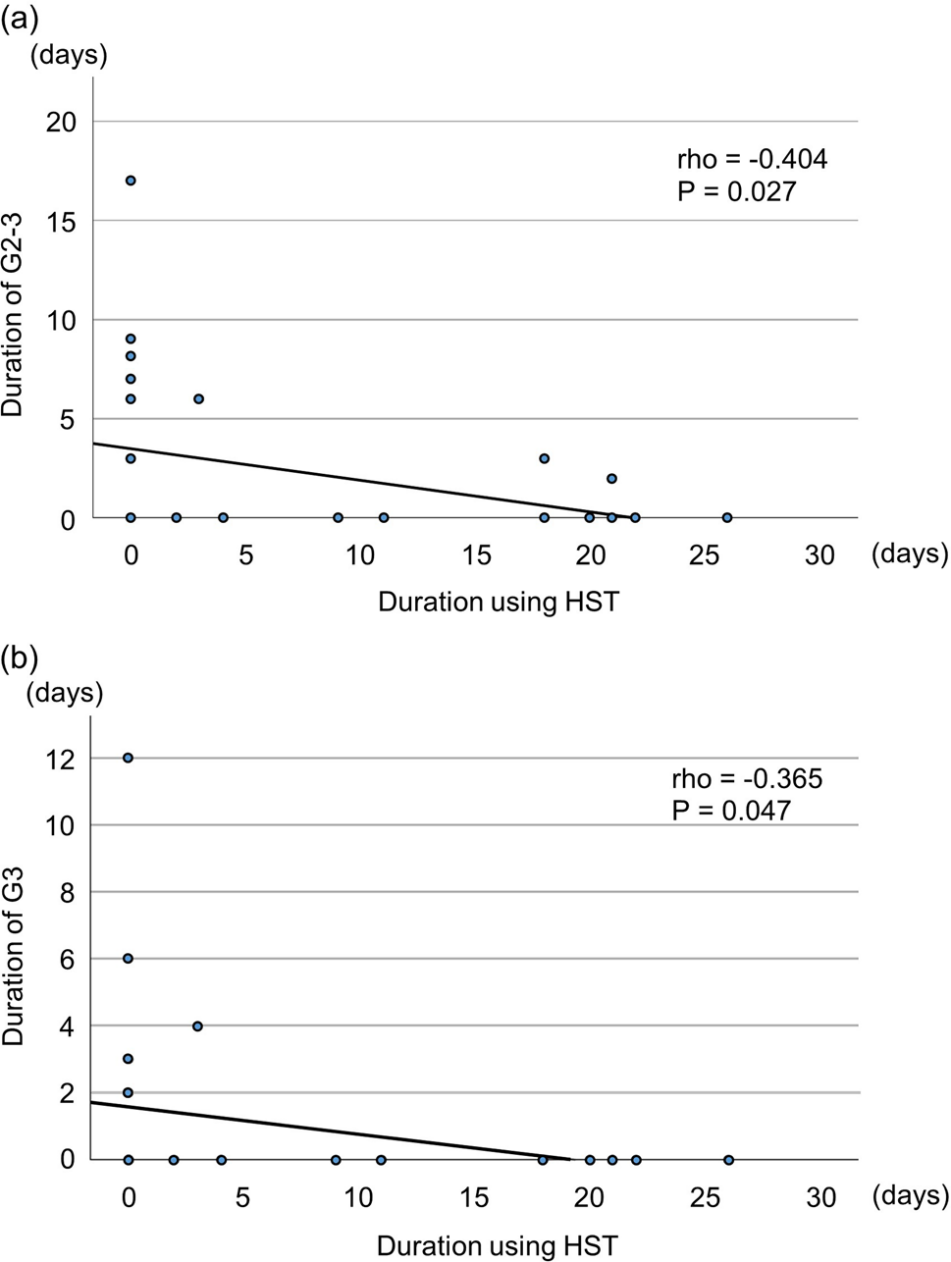
Correlation between duration of HST use and duration of oral mucositis. (**a)** Correlation between duration of HST use and duration of G2–3 oral mucositis. (**b)** Correlation between duration of HST use and duration of G3 oral mucositis. G, grade; HST, Hangeshashinto

Tables 3, 4, and 5 show the relationship of each variable with the duration of OM. The durations of G1–G3, G2–G3, and G3 OM were significantly longer in patients who received the MAC regimen than in those who received the RIC regimen (G1–G3 *P* = 0.001, G2–G3 *P* = 0.003, G3 *P* = 0.022). Moreover, there was a tendency toward a negative correlation between the duration of HST use and the duration of G2–G3 and G3 OM (G2–G3 correlation coefficient (rho) =  − 0.404, *P* = 0.027; G3 rho =  − 0.365, *P* = 0.047) (Tables 4 and 5; Fig. 2).

**Table 3:** Relationship of each variable with duration of G1–G3 oral mucositis

		G1–G3		
		Duration, days	Correlation coefficient	*P* value
(i) Categorical data				
Sex	Male	0 [0.0–13.0]		0.471^†^
	Female	7 [1.0–11.0]		
Smoking	( −)	5.5 [0.0–11.3]		0.836^†^
	( +)	6.5 [0.0–16.3]		
Drinking	( −)	5.5 [0.0–13.5]		0.755^‡^
	Socially	1 [0.0–10.0]		
	( +)	7 [3.0–14.0]		
Performance status	0	5 [0.0–8.0]		0.843^‡^
	1	6 [0.0–11.5]		
	2	7.5 [0.0, -]		
Conditioning regimen	MAC	11 [7.3–14.5]		0.001^†^
	RIC	0 [0.0–6.0]		
Donor	Related	3 [0.0–9.3]		0.056^†^
	Unrelated	13.5 [1.5–22.0]		
Alveolar bone loss ≥ 1/2	( −)	7 [0.0–12.0]		0.226^†^
	( +)	0 [0.0–6.0]		
Periodontal pocket depth ≥ 5 mm	( −)	6 [0.0–12.0]		0.666^†^
	( +)	1 [0.0–9.0]		
Bleeding on probing	( −)	10 [0.0–15.0]		0.112^†^
	( +)	1 [0.0–9.0]		
Periapical periodontitis ≥ 5 mm	( −)	6 [0.0–12.3]		0.801^†^
	( +)	6 [0.0–8.8]		
High-grade caries	( −)	6 [0.0–11.0]		0.980^†^
	( +)	4.5 [0.0–14.8]		
Tooth extraction/apicoectomy	( −)	7 [0.0–12.0]		0.650^†^
	( +)	6 [0.0–10.5]		
(ii) Continuous data				
Age			− 0.024	0.899^§^
Body mass index			− 0.193	0.307^§^
Duration from pretreatment to engraftment			0.155	0.414^§^
Number of remaining teeth			− 0.309	0.097^§^
Simplified debris index			− 0.240	0.202^§^
Duration of HST use			− 0.289	0.122^§^

Duration is presented as median [25–75% tale]

*G* grade, *MAC* myeloablative conditioning, *RIC* reduced-intensity conditioning, *HST* Hangeshashinto

^†^Mann–Whitney *U* test, ^‡^ Kruskal–Wallis test, ^§^Spearman’s rank correlation coefficient

**Table 4:** Relationship of each variable with duration of G2–G3 oral mucositis

		G2–G3		
		Duration, days	Correlation coefficient	*P* value
(i) Categorical data				
Sex	Male	0 [0.0–3.0]		1.000^†^
	Female	0 [0.0–6.0]		
Smoking	( −)	0 [0.0–6.0]		1.000^†^
	( +)	0 [0.0–3.0]		
Drinking	( −)	0 [0.0–6.3]		0.309^‡^
	Socially	0 [0.0–0.0]		
	( +)	0 [0.0–5.0]		
Performance status	0	0 [0.0–3.0]		0.770^‡^
	1	0 [0.0–5.0]		
	2	3 [0.0, -]		
Conditioning regimen	MAC	4.5 [0.5–7.0]		0.003^†^
	RIC	0 [0.0–0.0]		
Donor	Related	0 [0.0–2.3]		0.070^†^
	Unrelated	4.5 [0.0–8.5]		
Alveolar bone loss ≥ 1/2	( −)	0 [0.0–6.0]		0.386^†^
	( +)	0 [0.0–0.0]		
Periodontal pocket depth ≥ 5 mm	( −)	0 [0.0–3.0]		0.631^†^
	( +)	0 [0.0–7.0]		
Bleeding on probing	( −)	2 [0.0–6.0]		0.268^†^
	( +)	0 [0.0–3.0]		
Periapical periodontitis ≥ 5 mm	( −)	0 [0.0–3.8]		0.447^†^
	( +)	1.5 [0.0–6.0]		
High-grade caries	( −)	0 [0.0–3.0]		0.860^†^
	( +)	0 [0.0–9.5]		
Tooth extraction/apicoectomy	( −)	0 [0.0–6.0]		0.680^†^
	( +)	0 [0.0–3.0]		
(ii) Continuous data				
Age			− 0.049	0.796^§^
Body mass index			− 0.001	0.996^§^
Duration from pretreatment to engraftment			0.139	0.465^§^
Number of remaining teeth			− 0.288	0.123^§^
Simplified debris index			− 0.048	0.803^§^
Duration of HST use			− 0.404	0.027^§^

Duration is presented as median [25–75% tale]

*G* grade, *MAC* myeloablative conditioning, *RIC* reduced-intensity conditioning, *HST* Hangeshashinto

^†^Mann–Whitney *U* test, ^‡^ Kruskal–Wallis test, ^§^Spearman's rank correlation coefficient

**Table 5:** Relationship of each variable with duration of G3 oral mucositis

		G3		
		Duration, days	Correlation coefficient	*P* value
(i) Categorical data				
Sex	Male	0 [0.0–0.0]		0.767^†^
	Female	0 [0.0–2.0]		
Smoking	( −)	0 [0.0–0.5]		0.765^†^
	( +)	0 [0.0–0.0]		
Drinking	( −)	0 [0.0–2.3]		0.304^‡^
	Socially	0 [0.0–0.0]		
	( +)	0 [0.0–1.5]		
Performance status	0	0 [0.0–0.0]		0.480^‡^
	1	0 [0.0–0.0]		
	2	2 [0.0, -]		
Conditioning regimen	MAC	1 [0.0–3.8]		0.022^†^
	RIC	0 [0.0–0.0]		
Donor	Related	0 [0.0–0.0]		0.298^†^
	Unrelated	0 [0.0–5.0]		
Alveolar bone loss ≥ 1/2	( −)	0 [0.0–0.0]		0.886^†^
	( +)	0 [0.0–0.0]		
Periodontal pocket depth ≥ 5 mm	( −)	0 [0.0–0.0]		0.631^†^
	( +)	0 [0.0–3.0]		
Bleeding on probing	( −)	0 [0.0–2.0]		0.703^†^
	( +)	0 [0.0–0.0]		
Periapical periodontitis ≥ 5 mm	( −)	0 [0.0–0.0]		0.730^†^
	( +)	0 [0.0–2.3]		
High-grade caries	( −)	0 [0.0–0.0]		0.494^†^
	( +)	0 [0.0–5.3]		
Tooth extraction/apicoectomy	( −)	0 [0.0–1.0]		0.773^†^
	( +)	0 [0.0–0.0]		
(ii) Continuous data				
Age			− 0.141	0.457^§^
Body mass index			− 0.073	0.702^§^
Duration from pretreatment to engraftment			0.171	0.367^§^
Number of remaining teeth			− 0.133	0.485^§^
Simplified debris index			0.068	0.720^§^
Duration of HST use			− 0.365	0.047^§^

Duration is presented as median [25–75% tale]

*G* grade, *MAC* myeloablative conditioning, *RIC* reduced-intensity conditioning, *HST* Hangeshashinto

^†^Mann–Whitney *U* test, ^‡^ Kruskal–Wallis test, ^§^Spearman’s rank correlation coefficient

## Discussion

In this single-center study, the effects of HST for prevention of OM were investigated in patients undergoing HSCT. The incidence of OM was not significantly different between the HST group and control group. However, the duration of HST use showed a tendency toward a negative correlation with the duration of ≥ G2 OM.

OM is a common adverse event in patients with cancer. Vagliano et al. [16] reported that 71.4% of patients undergoing HSCT developed OM and that 21.6% had ≥ G3 OM. Guberti et al. [17] found that 78.0% of patients who underwent HSCT developed OM. In a systematic review, the incidence of OM in patients undergoing HSCT was 83.5% among seven studies [2]. In the present study, 60.0% of patients developed OM. Although not definitive because of the small number of patients, this incidence is likely lower than that in previous reports. In this study, the use of concomitant drugs or combination therapies was not limited. Moreover, oral care education and professional oral care interventions by dentists and dental hygienists were given to all patients throughout the study period. These factors may have contributed to better oral health and reduction of OM in this study.

Symptoms of OM were recognized from 0 to 19 days after starting the conditioning regimen and continued from 1 to 23 days during the study period (data not shown). Onset and progression of OM are related to various factors, such as the presence of other disorders, the patient’s general condition, and oral health [18]. Pathobiologically, onset of the first phase of OM occurs immediately after starting chemotherapy, and the healing phase usually occurs approximately 2 to 4 weeks after the final day of chemotherapy [18–20]. Clinically, the mean onset of OM was 9.1 days after initiation of the conditioning regimen and that the duration was 10.4 days in patients undergoing HSCT [17]. Onset and healing of OM in this study indicated in accordance with these reports.

OM was recognized in all patients who received the MAC regimen in this study. By contrast, 33.3% of patients who received the RIC regimen developed OM. In addition, no patient who received the RIC regimen developed G3 OM. There was a significant difference in the incidence of G1, G2, and G3 OM between the MAC and RIC regimens. Moreover, the duration of G1–3, G2–3, and G3 OM was significantly longer in the MAC than RIC regimen. One systematic review showed that the RIC regimen was associated with a high incidence of OM similar to that associated with the MAC regimen [21]. The authors of the review also noted that various definitions were used to categorize patients into the MAC and RIC regimens. In the Nagasaki Transplant Group, the conditioning intensity is determined by the consensus of the Center for International Blood and Marrow Transplant Research criteria [22, 23]. Several reports have shown that the appearance of OM with chemotherapy depends on the dose and type of chemotherapy and that the incidence of OM is higher with MAC than RIC regimens in patients undergoing HSCT [24, 25]. These findings are in agreement with our results.

In this study, the duration of HST use showed a negative correlation with the duration of both G2–3 and G3. Kono et al. [13] demonstrated that use of HST had therapeutic effects on chemotherapy-induced OM in patients with advanced colorectal cancer. Matsuda et al. [14] reported that the duration of ≥ G2 OM was significantly reduced when HST was used in patients who received chemotherapy for colorectal cancer. Biologically, chemotherapy induces DNA damage of stem cells in the basal layer of the submucosa; consequently, mucosal tissues are broken down and become ulcerated [9, 10, 12]. Reactive oxygen species are formed in response to mucosal damage, and release of proinflammatory cytokines is induced. Furthermore, chemotherapy reduces immunity; accordingly, oral bacteria colonize the ulcerated mucosal surface, the injury is potentiated, and infection develops. The components of HST have effective anti-oxidation, anti-inflammatory, and anti-bacterial effects [11, 12]. Although damage of mucosal tissue is considered unavoidable during chemotherapy, our study data suggest that the ingredients in HST contributed to suppression of OM deterioration.

No patients in the control group dropped out of this study. However, six patients in the HST group withdrew from the study because of nausea (*n* = 4), fever (*n* = 1), or a bitter taste when using the mouthwash (*n* = 1) (data not shown). Whether the nausea was secondary to HST use in this study remains unclear, because nausea is a known side effect of cancer chemotherapy. HST has a notably unique taste that is characterized by bitterness. In addition, potential adverse effects of HST include interstitial pneumonia and pseudohyperaldosteronism. However, the use of HST as a mouthwash solution as in the present study is considered safe and minimally concerning. Initially, we considered comparing the data between the HST group and control group. However, the duration of HST use was variable among the patients who discontinued HST use, ranging from 2 to 11 days. In addition, according to the study protocol, we did not exclude the data of the patients who discontinued HST use. Therefore, we determined that the effects of HST would be more accurately verified by the period of HST use than by whether HST was used (Tables 2, 3, 4 and 5; Fig. 2).

This study had three main limitations. First, the sample size was small, and all participants who withdrew from the study were in the HST group. Second, we may not have been able to completely prevent placebo effects and bias because the study was not double-blinded. Third, we did not restrict the use of concomitant medications for stomatitis, such as steroid treatments and anti-inflammatory gargle solutions. HST has a known tendency to inhibit OM exacerbation, but further clinical and biological studies are necessary for a full understanding of inhibition of OM in patients undergoing HSCT.

## Data Availability

All data are available on request.
